# Extraordinary Fecundity

**Published:** 1852-10

**Authors:** 


*Extraordinary Fecundity.*

A Belgian paper states that a woman, thirty-three years of age, is now living at Liege, who affords an astonishing example of fecundity. She was lately confined of triplets, who are respectively her twenty-second, twenty-third, and twenty-fourth children. She has thus had, during nine years of married life, twenty-four children, all in good health, and of the female sex.—*Ibid.*

